# Biocompatibility Evaluation of Novel Experimental Titanium Alloys for Dental Implants

**DOI:** 10.3390/dj14010006

**Published:** 2025-12-22

**Authors:** Vlad-Gabriel Vasilescu, Lucian Toma Ciocan, Andreea Mihaela Custura, Miruna Stan, Florin Miculescu, Cosmin Mihai Cotrut, Diana Maria Vranceanu, Elisabeta Vasilescu, Marina Imre, Silviu Mirel Pițuru

**Affiliations:** 1 Discipline of Dental Prosthesis Technology, Faculty of Dentistry, “Carol Davila” University of Medicine and Pharmacy, Dionisie Lupu Street, No. 37, District 2, 020021 Bucharest, Romania; vlad.vasilescu@umfcd.ro; 2 Department of Biochemistry and Molecular Biology, Faculty of Biology, University of Bucharest, 9195 Splaiul Independentei, 050095 Bucharest, Romania; miruna.stan@bio.unibuc.ro; 3 Faculty of Materials Science and Engineering, National University of Science and Technology POLITEHNICA Bucharest, Splaiul Independentei 313, J Building, 060042 Bucharest, Romania; florin.miculescu@upb.ro (F.M.); cosmin.cotrut@upb.ro (C.M.C.); diana.vranceanu@upb.ro (D.M.V.); 4 General Association of Engineers in Romania (AGIR), AGIR Board of Directors, Victoriei Boulevard 118, 030167 Bucharest, Romania; elisabeta.vasilescu@yahoo.com; 5 Discipline of Prosthodontics, Faculty of Dentistry, “Carol Davila” University of Medicine and Pharmacy, 37 Dionisie Lupu Street, District 2, 020021 Bucharest, Romania; marina.imre@umfcd.ro; 6 Department of Organization, Professional Legislation and Management of the Dental Office, Faculty of Dental Medicine, “Carol Davila” University of Medicine and Pharmacy, 17-23 Plevnei Str., 020021 Bucharest, Romania; silviu.pituru@umfcd.ro

**Keywords:** dental implants, new Ti alloys, corrosion rate, biocompatibility, human osteoblasts, human gingival fibroblasts, MTT test, cell viability

## Abstract

**Background/Objectives**: The purpose of this study was to assess the in vitro biocompatibility and corrosion resistance of five titanium alloys that have been recently developed for dental implant applications, whose compositions were designed to align with current approaches in the development of novel biomaterials. Priority was given to limiting the harmfulness associated with specific chemical elements present in common conventional alloys and increasing corrosion resistance to improve the biomaterial–tissue cellular interaction. **Methods**: For this purpose, five types of titanium alloys with original chemical compositions (Ti1–Ti5) were developed. The electrochemical behavior of the alloys was analyzed by evaluating the corrosion resistance in environments that simulate the oral environment, as well as the cellular behavior, by evaluating the viability, growth, and proliferation of human cells on osteoblasts and gingival fibroblasts. Detailed analysis of the chemical composition by scanning electron microscope (SEM/EDS) methods was used. The corrosion rate of the alloys in artificial saliva was tested using the polarization resistance technique (Tafel). Human osteoblasts (hFOB cell line) and human gingival fibroblasts (hFIB-G cell line) were used to measure biocompatibility in vitro. **Results**: The Ti5 alloy demonstrated the highest cell viability and the lowest corrosion rate (0.114 μm/year) among all tested compositions, with the Ti3 alloy containing Mo and Zr following closely behind. The Ti2 alloy exhibited reduced biocompatibility because of the inclusion of Ni and Fe in its composition. **Conclusions:** Taken together, the results of this study provide useful information on the basic characteristics of titanium alloys with original chemical compositions. The titanium alloys were analyzed in comparison with common conventional alloys (Cp–Ti and Ti6Al4V) as well as alloys such as Ti–Zr, Ti–Nb, and Ti–Nb–Zr–Ta, which are considered to be viable alternatives to conventional materials for making dental implants.

## 1. Introduction

The successful selection and use of materials in oral implantology requires the continuous evaluation of the factors that contribute to ensuring implant durability, from the nature of the biomaterial to the characteristics of the optimally designed biosurface.

It is estimated that 70–80% of biomedical implants are made of metal materials [1,2]. Their mechanical qualities make these metallic materials extremely valuable. However, their biological compatibility has become a primary study focus, as the contact between an implant and the adjacent tissues must not provoke responses that jeopardize stability or osseointegration.

Research aimed at developing new bioalloys for dental implants focuses on designing compositions that ensure optimal tissue biocompatibility [3,4,5,6,7,8,9,10,11,12].

The oral environment is chemically unfavorable due to frequent and substantial pH variations. Metallic materials exhibiting inadequate chemical stability may therefore dissolve, degrade, erode, or corrode over time.

Among these, corrosion is the most undesirable reaction between a metallic material and the oral environment. It generates toxic degradation products that may be released into adjacent tissues, where they can trigger unwanted cellular and extracellular reactions [13,14]. This is the reason why, in general, metallic biomaterials are characterized and classified based on their corrosion behavior as a determinant of their biocompatibility [15,16,17,18,19,20,21,22,23,24,25].

Metals and alloys used as implant materials are those that can form stable and protective passivity films on their surface in a corrosive environment. Of these, pure commercial titanium (Cp–Ti) and titanium alloys (frequently Ti6Al4V) are still the most widely used in dental implantology, being considered biocompatible in contact with bone and gingival tissues and capable of osseointegration [26,27,28].

Titanium is considered an inert material because, in contact with a tissue medium, it is rapidly inactivated by the formation of a thin layer of oxides (monoxide, dioxide, and trioxide). The thin, tenacious, and protective titanium oxide film formed in less than a second ensures good corrosion resistance. In the case of titanium alloys that have proven good corrosion resistance, the oxide layer formed can be thicker, very stable, and even better tolerated than pure titanium (Cp–Ti).

The Ti6Al4V alloy (α + β) acts as a strong and corrosion–resistant substitute for Cp–Ti. However, it has potentially harmful components, like aluminum and vanadium. Upon release due to corrosion or wear, these ions might interfere with biological processes at the tissue–implant interface, undermining osseointegration and long–term stability [29,30,31,32,33,34,35,36,37].

Early research on the limitation of these effects focused on replacing vanadium with niobium in titanium alloys, leading to two new V–free (α + β) type Ti–based alloys, i.e., Ti–6Al–7Nb and Ti–5Al–2.5Fe, characterized by mechanical and corrosion resistance comparable to those of Ti6Al4V alloy [38,39]. Studies indicated good corrosion resistance and acceptable biological properties due to the absence of vanadium, with Ti–6Al–7Nb considered suitable for the manufacture of dental implants [40,41,42,43]. Also, in vitro studies have shown a cellular behavior (osteoblast proliferation) comparable to or even better than that of Cp–Ti. It is estimated that human gingival fibroblasts adhere and proliferate to similar degrees on both alloys, while the osseointegration capacity evaluated in animal models (dogs) is also good [36,44,45].

Relatively recent research attests to the fact that Ti–Zr binary alloys are a good alternative to conventional metals, such as Cp–Ti and titanium alloys frequently used today in dental implantology, although they have demonstrated a lower osseointegration capacity [46,47,48]. The improved corrosion resistance of these alloys is explained by the formation of a thicker and denser passive film of titanium oxide on the surface, reinforced by the presence of zirconium oxide (ZrO_2_). Among the Ti–Zr alloys, the one with practical utility properties confirmed by laboratory studies and clinical practices is the Ti15Zr alloy (Roxolid), although other compositional reports are still being investigated. Studies conducted in artificial saliva and Ringer’s solution 2–3 attest to the dependence of corrosion resistance of Ti–Zr alloys on the concentration of Zr in the composition of the alloy [49,50,51,52,53]. On the other hand, binary Ti–Nb alloys demonstrate improved corrosion behavior, although cellular behavior shows that the proliferation and growth of human fibroblasts are slower than on Cp–Ti [54,55].

The alloying of titanium with more than three elements is most often found in systems containing niobium, molybdenum, tantalum, and zirconium. These elements ensure biocompatibility and good osteoconductivity [56,57,58]. Quaternary alloys provide increased corrosion resistance relative to ternary and binary systems. Research on Ti–Nb–Zr–Ta alloys has demonstrated an absence of cytotoxicity towards osteoblastic cells, with cell proliferation similar to that of Ti–6Al–4V and a greater capacity for differentiation [59,60].

Current research in the field and recent approaches to developing new titanium alloys with non–cytotoxic elements have motivated studying the influence of chemical composition on the biocompatibility of alloys containing elements such as molybdenum, niobium, zirconium, and tantalum. The objective is to design bioalloy compositions with properties that ensure the biofunctionality of the implant while simultaneously providing high corrosion resistance and compatibility with living tissues.

## 2. Materials and Methods

Samples of five types of titanium alloys with original chemical compositions were used. Alloys contain non–toxic alloying elements capable of improving corrosion resistance and biocompatibility.

The samples were also analyzed by scanning electron microscopy (SEM/EDS) for morphology and chemical composition evaluation. Scanning electron microscopy (SEM) was performed with the equipment Thermo Fisher Quattro S (Thermo Fisher Scientific, Waltham, MA, USA).

The corrosion resistance evaluation tests were performed using a Potentiostat/Galvanostat (PARSTAT 4000, Princeton Applied Research, Oak Ridge, TN, USA), coupled to a low current module (LCI, Princeton Applied Research, Oak Ridge, TN, USA). The potentiodynamic curves were obtained using VersaStudio software (version 2.62, Princeton Applied Research, Oak Ridge, TN, USA). The evaluation of corrosion resistance in artificial saliva was performed by the polarization resistance technique (Tafel). Disc–shaped alloy specimens (Ø 5 mm) were individually metallographically prepared by grinding with SiC papers (grit 500–1000), polished using 1 μm grain size Al_2_O_3_ suspension, and subjected to ultrasonic cleaning in ethanol. An electrical wire was attached to the unpolished surface, and each specimen was covered in silicone, with the polished region remaining exposed for electrochemical analysis. For the tests, a corrosion cell was used, consisting of a saturated calomel electrode (SCE) as a reference electrode, a platinum electrode, an auxiliary electrode, and the working electrode consisting of the investigated samples. The tests were performed at human body temperature (37 ± 1 °C) using Fusayama–Meyer artificial saliva as an electrolyte, with the following chemical composition: 0.4 gL^−1^ NaCl, 0.9 gL^−1^ KCl, 1 gL^−1^ urea, 0.69 gL^−1^ NaH_2_PO_4_, 0.795 gL^−1^ CaCl*2H_2_O (pH 5.2). It should be noted that all chemicals were purchased from Sigma Aldrich, and electrochemical tests were performed according to the experimental procedure outlined in ASTM G59 of 1997 (reapproved in 2003). For the measurement of biocompatibility in vitro, human osteoblasts (hFOB 1.19 cell line, Cat. No. CRL-3602 (ATCC, Manassas, VA, USA)) were maintained at a temperature of 34 °C in a humidified atmosphere with 5% CO_2_, in Dulbecco Modified Eagle’s Medium (DMEM)/Ham’s F–12 medium without phenol red (1:1), supplemented with 10% fetal bovine serum (FBS), 2.5 mM L–glutamine, and 0.3 mg/mL antibiotic G418. Human gingival fibroblasts (hFIB–G cell line, Cat. No. 1110412 (Provitro AG, Berlin, Germany)) were cultured in DMEM with 10% FBS at 37 °C in an atmosphere humidified with 5% CO_2_. The cells were seeded at a density of 2 × 10^4^ cells/cm^2^ onto the tested samples, which were previously sterilized for 2 h under UV light, and onto the plastic surface of 6–well tissue culture plates, which served as the experimental control.

The biocompatibility measurement was performed after 48 h of incubation under the conditions described above. The release of lactate dehydrogenase (LDH) as a consequence of cell membrane integrity loss was quantified using the Cytotoxicity Detection KitPLUS (Roche, Basel, Switzerland) according to the manufacturer’s instructions. The procedure was as follows: 50 μL of culture medium was incubated with 100 μL of a mixture containing catalyst and dye at 25 °C for 30 min in the dark. After stopping the reaction, the absorbance was read at 490 nm.

In addition, the level of nitric oxide (NO) released into the culture medium due to inflammatory processes was measured with the Griess reagent. Briefly, 80 μL of culture medium was mixed with the same volume of Griess reagent, and absorbance was measured at 550 nm using a FlexStation 3 microplate reader (Molecular Devices, San Jose, CA, USA).

Cell viability was assessed by incubating samples with MTT solution (1 mg/mL) for 2 h at 37 °C, followed by solubilization of formazan crystals with 2–propanol and measurement of absorbance at 595 nm using a microplate reader. Actin filaments were observed by staining with 20 μg/mL FITC–phalloidin for one hour after the cells were fixed and permeabilized, and visualization was performed under the Olympus IX71 inverted fluorescence microscope (Olympus IX71, Olympus Corporation, Tokyo, Japan). Visualization of viable and non–viable cells was performed with the LIVE/DEAD™ test kit (L3224, Molecular Probes, Eugene, OR, USA) under the Olympus IX71 microscope after incubating cells with AM and EthD–1 calcein for 30 min at room temperature.

Statistical analysis: The results obtained on human cells were presented as the mean value ± standard deviation (SD) of three different experiments. Statistical analysis was performed using comparisons between groups assessed by single–direction ANOVA, followed by the post hoc Bonferroni test (GraphPad Prism software, version 5, GraphPad Software, Inc., La Jolla, CA, USA). Values below 0.05 were considered statistically significant. The normality of data distribution was verified using the Shapiro–Wilk test, and the homogeneity of variances was assessed using Levene’s test prior to applying one–way ANOVA. The sample size was selected based on previous biocompatibility studies using similar cell models, ensuring adequate statistical power to detect differences between control and Ti alloy samples.

## 3. Results

### 3.1. Compositional Analysis of Ti1–Ti5 Alloys

The characterization of the samples (Ti1–Ti5 alloys) was performed by scanning electron microscopy (SEM) and energy–dispersive spectroscopy (EDS); the results are presented in Table 1.

### 3.2. Evaluation of Corrosion Resistance in Artificial Saliva (Fusayama–Mayer) of Ti–Ti5 Alloys

The evaluation of corrosion resistance in artificial saliva was performed by the polarization resistance technique (Tafel technique). This technique consists of drawing linear polarization curves involving the following steps:

- Measurement of the open circuit potential (E_OC_) for a duration of 6 h;
- Plotting potentiodynamic polarization curves from −0.2 V (vs. E_OC_) to + 0.2 V (vs. E_OC_)—Tafel curves, with a scan rate of 0.167 mV/s.

The investigated samples were coded according to Table 2.

**Figure 1:**
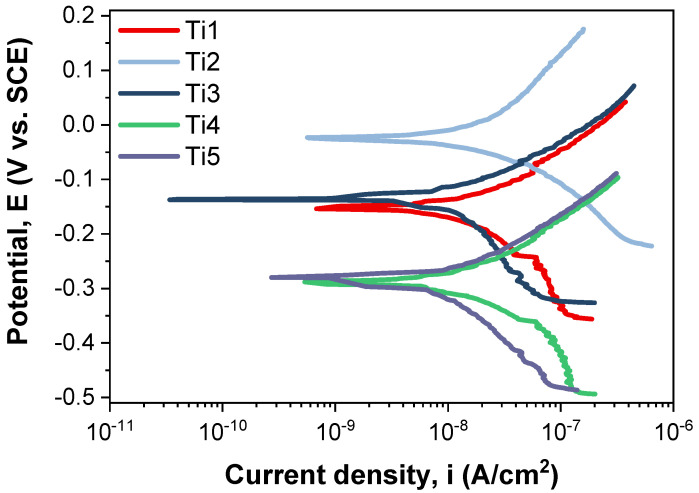
Tafel plots corresponding to the investigated samples.

The Tafel plots corresponding to the tested samples are shown in Figure 1.

The resistance to polarization was achieved according to ASTM G59–97 (2003) using the following formula:

(1) $$R_{p}=\frac{1}{2.3}\cdot \frac{\beta_{a}\beta_{c}}{\beta_{a}+\beta_{c}}\cdot \frac{1}{i_{cor}}$$

where

β_a_—the slope of the anodic curve;

β_c_—the slope of the cathodic curve;

i_corr_—the density of the corrosion current.

The corrosion rate was calculated according to ASTM G102–89 (2015) using the following calculation formula:

(2) $$CR=K_{i}\frac{i_{corr}}{\rho }EW$$

where CR—corrosion rate;

K_i_—3.27 × 10^−3^;

ρ—material density;

EW—the equivalent weight.

The corrosion resistance of a material can be examined on the basis of several evaluation criteria. In this light, it is widely known that an enhanced corrosion behavior is indicated by a more electropositive corrosion potential (E_corr_), increased polarization resistance (R_p_), a small corrosion current density (i_corr_), and a lower corrosion rate (CR), reflecting improved material stability and corrosion resistance.

If we consider the corrosion potential value (E_corr_), more electropositive E_corr_ corrosion potential values exhibit better electrochemical behavior. Comparing the values in Table 3, it can be observed that the T2 sample (−23.22 mV) has the most electropositive potential, followed by the T3 sample with a value of −128.72 mV.

Comparing the corrosion current density (i_corr_) obtained for the investigated samples, it can be noted that the lowest value of this parameter is obtained for the T5 sample (13.12 nA/cm^2^), closely followed by the T3 sample (17.47 nA/cm^2^).

It is known that a high polarization resistance (R_p_) highlights a good corrosion behavior of a material, and a low value of this parameter highlights a worse corrosion behavior. In this case, the highest value was recorded for the T5 sample (2959.94 kΩ × cm^2^), followed closely by the T3 sample (2424.37 kΩ × cm^2^). From the point of view of corrosion rate, the lowest value, which highlights a better corrosion behavior, was that of the T5 sample, which registered a value of 0.114 μm/year. The T3 sample followed closely behind, with a corrosion rate value of 0.197 μm/year.

Compared to pure titanium, which has a homogeneous microstructure that promotes the formation of a uniform and protective oxide layer, Ti–based alloys exhibit a microstructural architecture. This includes grain boundary segregation and phase inhomogeneity, which can promote the development of micro–galvanic cells. Such localized electrochemical activity accelerates corrosion processes and consequently diminishes the overall corrosion resistance of the alloy.

Another important factor influencing the corrosion behavior of Ti–based alloys is the material’s condition, which is determined by the manufacturing and processing methods employed. These methods can significantly affect the microstructural characteristics, such as phase distribution, grain size, and surface morphology, which in turn impact the formation and stability of the passive oxide layer and the alloy’s overall corrosion resistance [61].

### 3.3. In Vitro Biocompatibility Testing of Ti1–Ti5 Alloys

**Figure 2:**
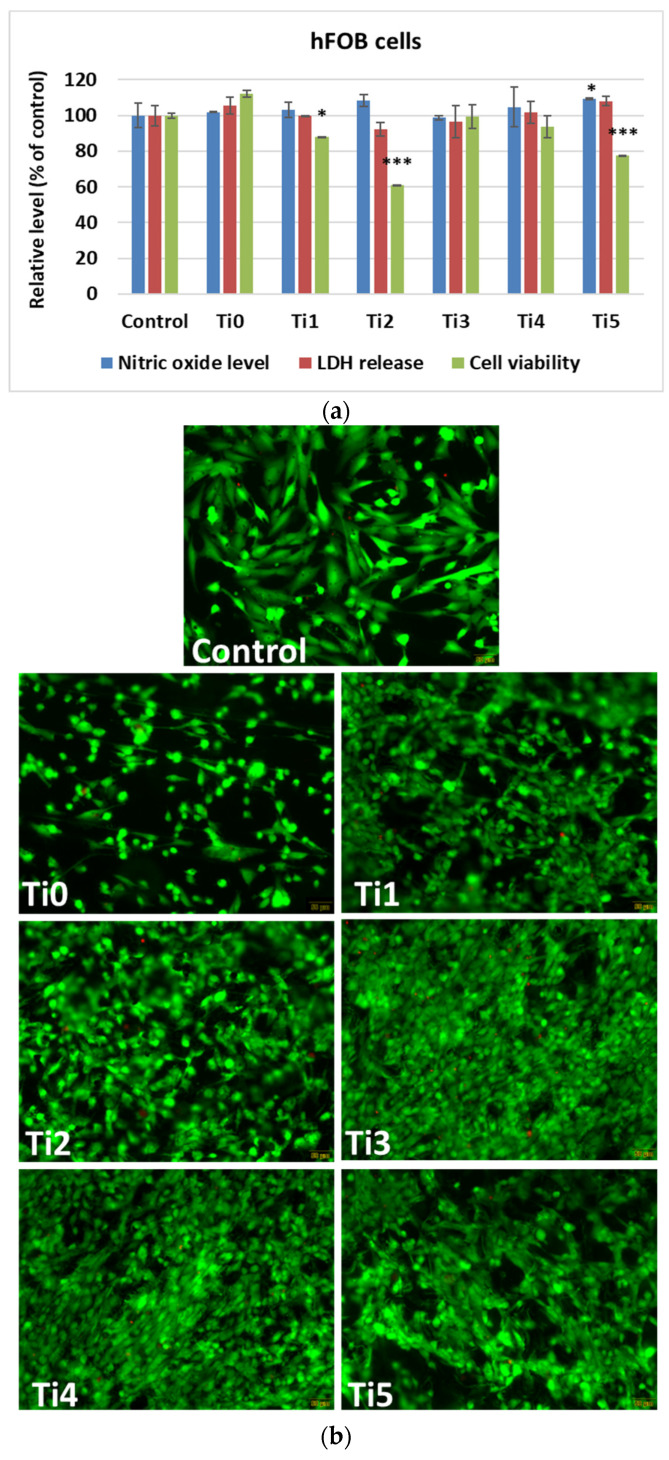
Biocompatibility of samples tested on human osteoblasts (hFOB 1.19 cells) measured after 48 h: (**a**) NO and LDH release levels and cell viability (results calculated as averages ± standard deviation of three independent experiments and presented relative to the control–free sample; * *p* < 0.05 and *** *p* < 0.001 compared to control), (**b**) staining of Live/Dead cells (green: live cells labeled with calcein AM; red: dead cells labeled with EthD–1; scale bar: 50 μm).

**Figure 3:**
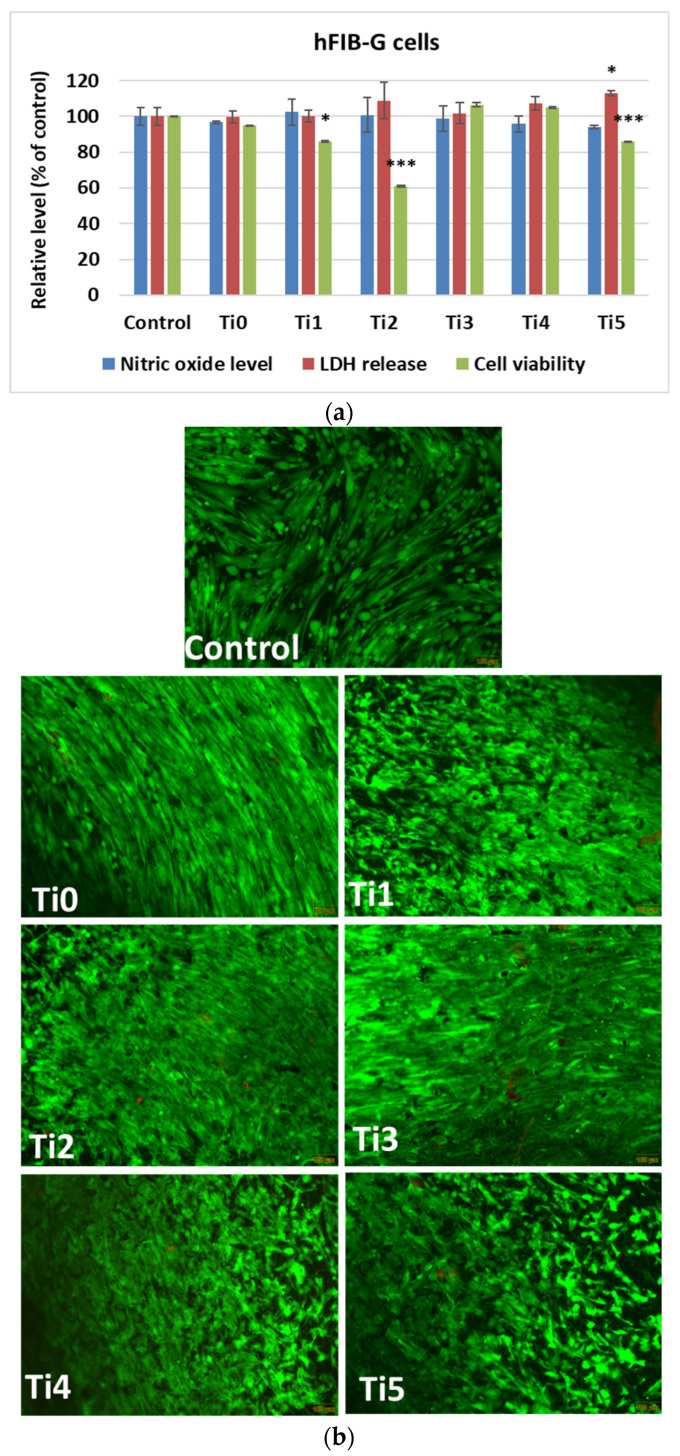
Biocompatibility of samples tested on human gingival fibroblasts (hFIB–G cells) after 48 h: (**a**) NO and LDH release levels, cell viability (results are calculated as means ± standard deviation of three independent experiments and presented relative to the sample–free control; * *p* < 0.05 and *** *p* < 0.001 compared to control), and (**b**) staining of Live/Dead cells (green: live cells labeled with calcein AM; red: dead cells labeled with EthD–1; scale bar: 100 μm).

**Figure 4:**
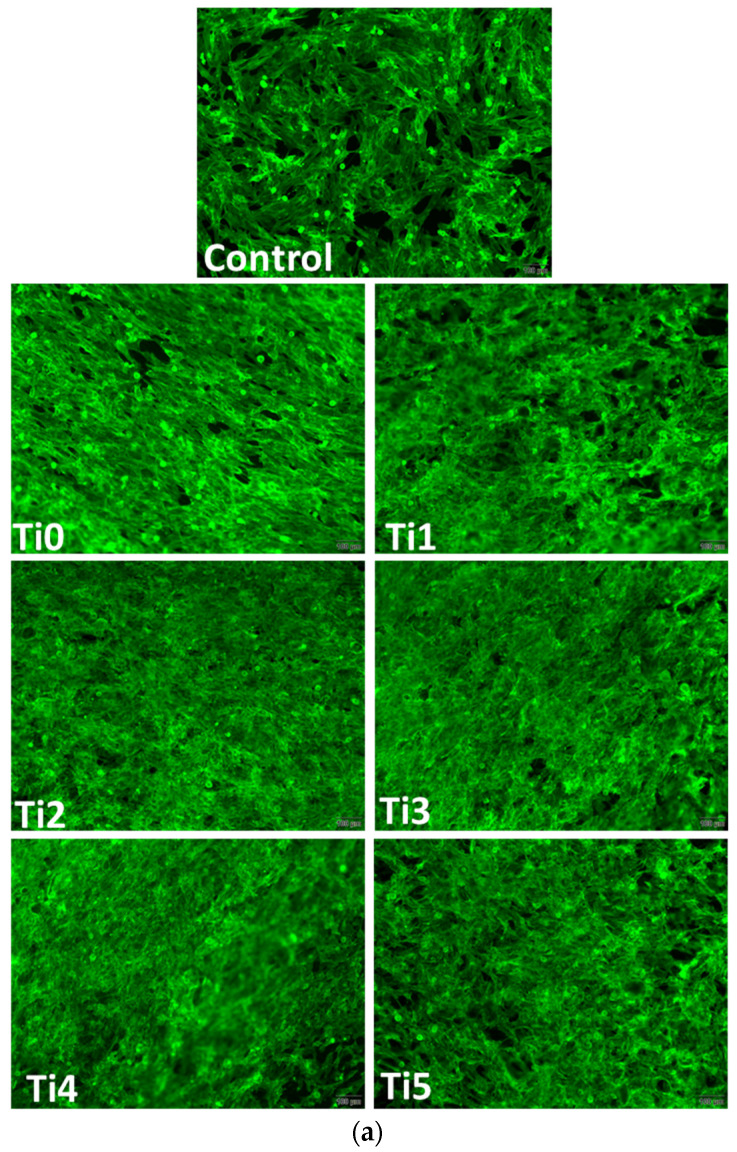

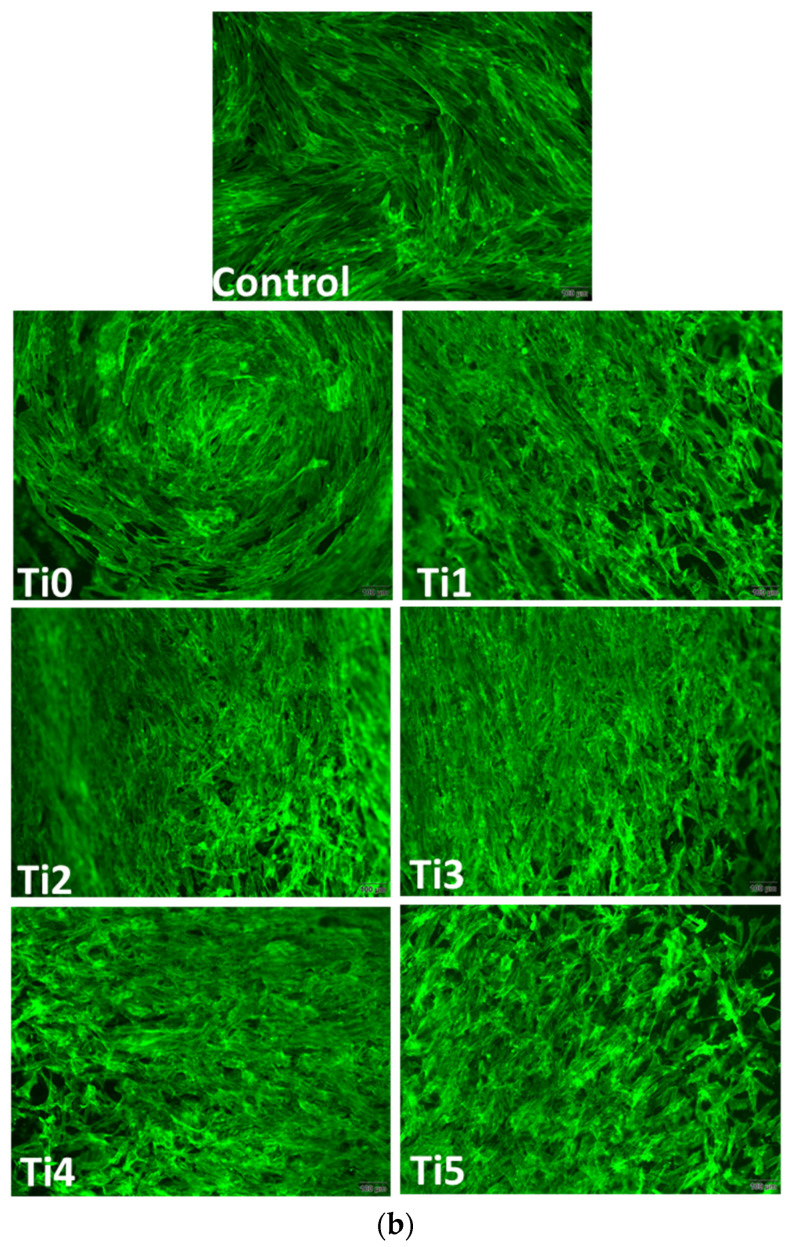
Staining of actin filament morphology (green: F–actin labeled with phaloidin–fluorescein isothiocyanate; blue: nuclei labeled with 4′,6–diamidino–2–phenylindole; scale bar: 100 μm) in (**a**) human osteoblasts (hFOB 1.19 cells) and (**b**) human gingival fibroblasts (hFIB–G cells) after 48 h of incubation.

In vitro results after 48 h of growth of human cells (osteoblasts—Figure 2, and gingival fibroblasts—Figure 3) on experimental alloys (encoded Ti1, Ti2, Ti3, Ti4, and Ti5) confirmed their biocompatibility, while cytoskeletal organization was further assessed by F-actin staining (Figure 4a,b). The Ti0 sample is taken as a reference, made of pure commercial titanium commonly used for dental implants. The MTT assay (Figure 2a and Figure 3a) indicated generally good metabolic activity on all tested alloys, with a significant decrease for Ti2 in both cell types compared to the control. The Live/Dead staining (Figure 2b and Figure 3b) confirmed these results, showing a higher proportion of non–viable cells on Ti2, while the remaining alloys supported cell viability comparable to the control.

LDH release and NO production values were similar to those of the control group, suggesting the absence of significant membrane damage or inflammatory response.

F–actin staining (Figure 4) revealed well–organized cytoskeletal structures in cells grown on Ti1, Ti3, Ti4, and Ti5 surfaces, indicating good adhesion and normal morphology. On Ti2, actin filaments appeared less organized, correlating with the reduced viability observed in MTT and Live/Dead assays. The altered cytoskeletal pattern was consistent with a lower level of biocompatibility for Ti2, most likely due to the presence of Ni and Fe in its composition, which may promote ion release and oxidative stress, affecting cell metabolism and adhesion.

## 4. Discussion

The development of new alloys with properties that increase the performance of conventional ones is a major goal in dental implantology. The most recent studies focus on alloys with high biocompatibility that facilitate the direct and functional connection between a vital bone and the implant inserted into this bone, which defines the osseointegration process. Achieving osseointegration and maintaining tissue integration of implants depends on the quality of the biomaterial, as assessed by corrosion resistance and cell behavior, which are basic criteria for their selection on a scientific basis. Characterized from this point of view, a synthetic picture of the alloys for implants highlights the following aspects:

Cp–Ti is superior to Co–Cr alloys and stainless steels, which typically contain some harmful elements such as Ni, Co, and Cr [3,31]. In saline solutions near neutral pH, the corrosion rate is generally extremely low, and there is no evidence of intergranular corrosion or cracking.

Although biocompatible, in vitro and in vivo, Ti^4+^ ions inhibit the in vitro osteoclastic activity and reduce osteoblastic protein synthesis [39].

The Ti6Al4V alloy type (α + β) has better corrosion resistance but has cytotoxic elements such as Al and V in its composition. The replacement of vanadium with niobium in titanium alloys leads to two new types of V–free alloys (α + β), namely Ti–6Al–7Nb and Ti–5Al–2.5Fe. They are characterized by corrosion resistance comparable to the Ti6Al4V alloy, cellular behavior comparable to or even better than Cp–Ti, and good osseointegration capacity, as demonstrated in animal (dog) models [40,41,42,43,44,45,46].

Ti–Zr binary alloys have improved corrosion resistance due to the formation of a thicker and denser passive layer of titanium oxide and zirconium oxide. The value of corrosion resistance is dependent on the Zr content in the composition of the alloy. Recent studies on the development of new biocompatible alloys as alternatives to conventional ones have focused on binary Ti–Zr alloys. Although the osseointegration capacity of implants made from Ti–Zr alloys has been shown to be lower than that of commonly used alloys, binary Ti–Zr alloys exhibit improved corrosion resistance due to the formation of a thicker and denser passive titanium oxide layer, reinforced by zirconium oxide. Qian Zhao reports that “the enhanced corrosion resistance of these alloys can be attributed to the fact that Zr is an anodic alloying element for Ti, which directly reduces anodic activity” [10]. It was demonstrated that the corrosion resistance value depends on the Zr content within the alloy composition.

Cordeiro et al. showed that corrosion resistance can be correlated with alloy hardness, also highlighting the role of surface treatments (such as double acid etching) in increasing the stability of the protective oxide film [62]. Ho et al. and Hsu et al., who investigated Ti–Zr alloys with various zirconium concentrations (10%, 20%, 30%, and 40%), demonstrated that Ti–Zr with 40% Zr exhibits the highest hardness, flexural strength, elastic recovery capacity, and corrosion resistance, taking into account the phase structure of the alloys [57,63,64].

Cui et al. emphasized the role of surface treatments and demonstrated that thermal oxidation leads to an increase in the thickness and compactness of the formed oxide layer (ZrO_2_, ZrTiO_4_), providing high stability in fluoride–containing oral environments and in acidic media, significantly reducing the risk of pitting corrosion [65].

Mareci, Bolat, and collaborators tested the corrosion resistance of Ti–Zr alloys (55%, 75%, and 95% Zr) under various conditions and showed that Ti–Zr (55% Zr) exhibited the best corrosion resistance in artificial saliva and Ringer solutions [20,48]. It was demonstrated that the trend of improved corrosion resistance is reversed when testing in environments with high chloride ion content or high fluoride ion concentrations. However, regardless of the testing medium, it has been consistently shown that surface treatments significantly enhance the corrosion resistance of Ti–Zr alloys. For example, double acid etching improves the stability of the oxide layer, while thermal oxidation increases hardness and reduces wear rate.

Overall, controlled modifications of the implant biosurface aimed at improving its functional characteristics have led to remarkable results, which should be further leveraged to increase success rates in oral implantology [66,67,68,69,70,71,72,73,74,75,76,77,78,79,80].

Ti–Nb binary alloys demonstrate improved corrosion behavior, despite cellular behavior showing that the proliferation and growth of human fibroblasts is slower than on Cp–Ti [54,55]. Quaternary titanium alloys have been shown to have better corrosion resistance than commercial ternary alloys and binary alloys and a cellular behavior that exhibits comparable cell proliferation but greater cell differentiation than Ti–6Al–4V alloy [59,60].

As for the experimental Ti1–Ti5 alloys subjected to analysis for corrosion resistance evaluation, they demonstrated a varied corrosion behavior, dependent on chemical composition. The results emphasize the influence of harmful elements present in the composition of Ti2 (Fe and Ni), which also exhibit the highest measured corrosion rate (about 0.3 μm/year).

The lowest corrosion rate and, therefore, the better corrosion behavior was observed for the sample with the T5 composition, followed closely by the sample with the T3 composition (Zr and Mo). The measured values of the electrochemical parameters are consistent with the observed cellular behavior of the experimental alloys.

In human osteoblasts, the MTT test demonstrated generally good metabolic activity on most titanium surfaces, with a notable decrease in the case of the Ti2 composition sample, indicating reduced cell viability (Figure 2a). This was also confirmed by the Live/Dead staining (Figure 2b), which showed a higher proportion of non–viable cells on this alloy compared to the others. Ni and Fe are known to undergo partial ion release under physiological conditions, generating reactive oxygen species (ROS) and interfering with mitochondrial metabolism. Ni^2+^ can bind to cellular proteins and DNA, altering enzymatic activity, while Fe^2+^ can catalyze Fenton–type reactions, increasing oxidative stress. These processes can disrupt cytoskeletal integrity and reduce metabolic activity, explaining the lower cell viability and altered F–actin morphology observed on the Ti2 alloy surface. In contrast, the remaining surfaces supported the viability and function of osteoblasts at levels comparable to those in plastic control samples. The level of LDH release was almost comparable to that of the control samples, suggesting that there was no increased membrane damage and cytotoxicity for the Ti–based samples. In addition, nitric oxide (NO) levels were not altered compared to those in the control samples, potentially reflecting the absence of a stress response or inflammatory activation.

For human gingival fibroblasts, a similar trend was observed (Figure 3a). The MTT assay indicated a low cell viability after incubation on the Ti2 sample, while the other materials maintained values close to the control ones. Live/Dead (Figure 3b) staining confirmed a higher proportion of dead cells on the surface of Ti2. Both NO levels and LDH release remained at control values for all tested materials, confirming good biocompatibility.

F–actin staining (Figure 4), used to assess cytoskeleton integrity and cell adhesion, revealed well–spread cells with intact actin filaments on most surfaces tested in both cell lines. However, on the Ti2 surface, a slightly altered morphology of the actin architecture was observed for both osteoblasts and fibroblasts. These cytoskeletal changes could indicate impaired Ti2 surface biocompatibility, in accordance with viability data.

Alterations in F–actin organization indicate compromised cell adhesion and spreading, which are critical for successful osseointegration. The well–organized actin cytoskeleton observed on Ti3, Ti4, and Ti5 surfaces suggests stable focal adhesions and favorable cell–material interactions, promoting extracellular matrix deposition and tissue integration. In contrast, the disrupted actin pattern on Ti2 implies weaker adhesion forces and reduced mechanical stability at the implant–tissue interface, consistent with its higher cytotoxicity.

Molybdenum (Mo) contributes to corrosion resistance by stabilizing the passive oxide film on titanium surfaces and reducing pitting susceptibility in chloride–rich environments such as saliva. The Mo^6+^ ions incorporated into the Ti matrix improve the film density and stability, limiting ion diffusion and metal dissolution. Zirconium (Zr) enhances both corrosion resistance and cell compatibility through the formation of a compact Zr–enriched surface, which increases surface passivity and hydrophilicity, thereby promoting protein adsorption and osteoblast adhesion. The synergistic effect of Mo and Zr explains the low corrosion rate and favorable cellular response of the Ti3 alloy.

When analyzed in comparison with the materials commonly used for dental implants (Cp–Ti grade 4 and Ti6Al4V), the experimental alloys (Ti1–Ti4) exhibit mechanical strength properties (Rm, Rp, and HV hardness) with values significantly higher than those of commercially pure titanium (Cp–Ti grade 4), with the exception of Ti5. The plasticity–ductility properties are comparable to those of Cp–Ti grade 4 (Ti4 and Ti2 compositions) and to those of Ti6Al4V (Ti1 composition). All experimental alloys (Ti1–Ti4) present a lower elastic modulus (E) (78.5–97.5 GPa) compared with Cp–Ti grade 4 (102–104 GPa) and Ti6Al4V (113 GPa).

Considering the aspects related to corrosion behavior and biological compatibility (Ti2 may be less suitable due to the presence of elements such as Ni, Al, V, and Fe), the Ti3 alloy stands out as a promising candidate with potential indications for specific clinical situations in dental implantology.

## 5. Conclusions

This study addresses a topic intensively explored in recent years: the development of new titanium alloys capable of improving implant–tissue interactions, reducing post–implant healing time, and increasing the longevity of dental implants. Five alloys were investigated, each designed using non–allergenic and non–toxic elements (Nb, Ta, Zr, and Mo). The research focused on examining the relationship between alloy composition and biological compatibility, assessed through corrosion resistance tests and the evaluation of cellular behavior in human osteoblasts and gingival fibroblasts.

Corrosion resistance, evaluated through corrosion potential, corrosion current density, and polarization resistance, showed that alloy T3 (Mo and Zr) exhibited the lowest corrosion rate, with values comparable to T5 (commercially pure Ti). Thus, Ti3 demonstrated the most favorable corrosion behavior in the Fusayama–Maier environment.

In vitro analyses confirmed that the alloys generally supported good cellular responses, similar to commercially pure titanium used as the reference. However, the Ti2 alloy showed reduced viability in both osteoblasts and gingival fibroblasts. The MTT assay indicated decreased metabolic activity on Ti2, while the remaining materials maintained values close to the control. Live/Dead staining supported these findings by revealing a higher proportion of non–viable cells on the Ti2 surface.

LDH release remained at levels comparable to the control samples, suggesting no increased membrane damage or cytotoxicity for any of the tested alloys. Likewise, nitric oxide (NO) levels were unchanged relative to controls, indicating the absence of cellular stress or inflammatory activation. Together, LDH and NO measurements confirmed the overall biocompatibility of the materials.

Overall, the results provide valuable insights into the development of new titanium alloys for dental implants, highlighting the influence of composition on their biological performance. Among the investigated materials, the T3 alloy demonstrated the most advantageous behavior.

## Figures and Tables

**Table 1:** SEM and EDS analysis of Ti–Ti5 experimental alloys.

Alloy	SEM	EDS				
Ti1	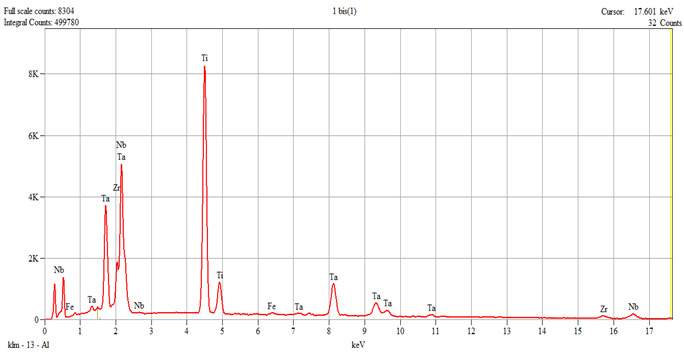 Live Time: 30.0 s.	Quantitative Results				
		Elements	Weight, %	Weight,% Error	Atom %	Atom, % Error
		Ti	39.60	±0.21	61.75	±0.32
		Fe	0.56	±0.06	0.75	±0.08
		Zr	9.64	±0.98	7.89	±0.81
		Nb	22.70	±1.05	18.25	±0.85
		Ta	27.50	27.50	±0.55	11.35
		Total	100.00		100.00	
Ti2	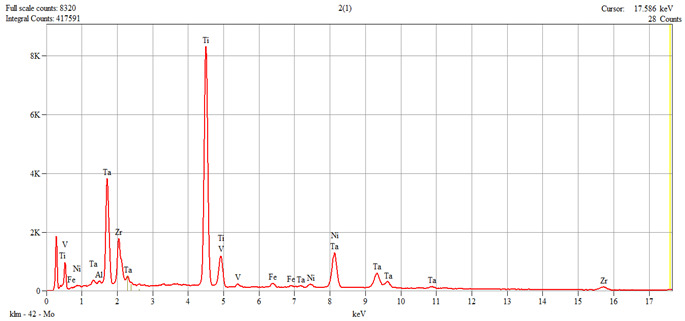 Live Time: 30.0 s.	Quantitative Results				
		Elements	Weight, %	Weight,% Error	Atom %	Atom, % Error
		Al	0.71	±0.08	1.89	±0.22
		Ti	44.16	±0.24	67.06	±0.36
		V	0.91	±0.12	1.30	±0.17
		Fe	1.03	±0.13	1.34	±0.16
		Ni	1.29	±0.08	1.59	±0.10
		Zr	15.05	±1.09	12.00	±0.87
		Ta	36.85	±0.64	14.81	±0.26
		Total	100.00		100.00	
Ti3	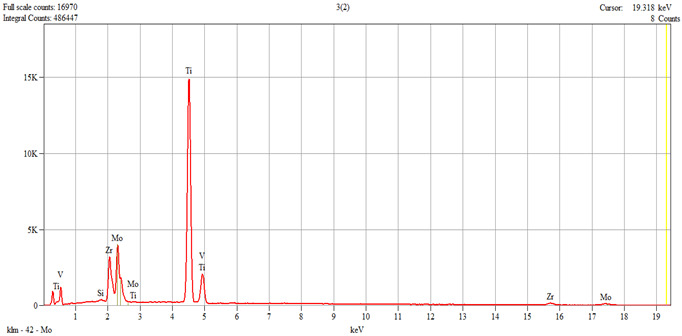 Live Time: 30.0 s.	Quantitative Results				
		Elements	Weight, %	Weight,% Error	Atom %	Atom, % Error
		Si	0.12	±0.03	0.23	±0.06
		Ti	66.88	±0.29	79.25	±0.35
		V	1.04	±0.11	1.16	±0.13
		Zr	14.41	±0.80	8.97	±0.49
		Mo	17.55	±1.28	10.38	±0.76
		Total	100.00		100.00	
Ti4	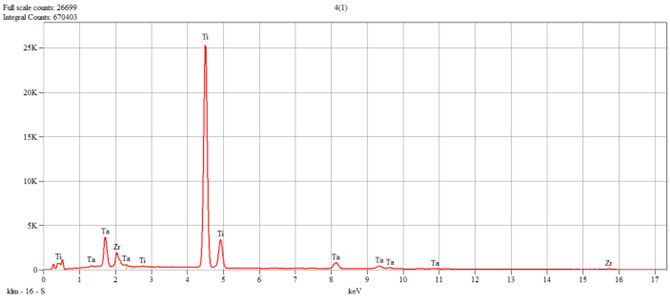 Live Time: 30.0 s.	Quantitative Results				
		Elements	Weight, %	Weight,% Error	Atom %	Atom, % Error
		Ti	78.76	±0.30	91.69	±0.35
		Zr	5.81	±0.50	3.55	±0.31
		Ta	15.43	±0.39	4.76	±0.12
		Total	100.00		100.00	
Ti5	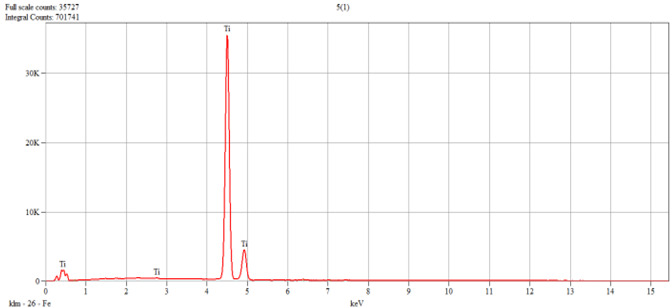 Live Time: 30.0 s.	Quantitative Results				
		Elements	Weight, %	Weight,% Error	Atom %	Atom, % Error
		Ti	100.00	±0.36	100.00	±0.36
		Total	100.00		100.00	

**Table 2:** Samples codification subjected to electrochemical tests.

Number	Samples Codification	Description
1	T1	Ti Alloy
2	T2	Ti Alloy
3	T3	Ti Alloy
4	T4	Ti Alloy
5	T5	Ti

**Table 3:** Electrochemical parameters when testing the corrosion rate of T1–T5 alloys.

Samples	E_corr_ (mV)	i_corr_ (nA/cm^2^)	β_c_ (mV)	β_a_ (mV)	R_p_ (kΩ × cm^2^)	CR (μm/year)
**T1**	−151.98	28.06	305.57	170.15	1693.30	0.217
**T2**	−23.22	36.61	181.18	323.59	1379.16	0.295
**T3**	−128,72	17.47	316.72	140.78	2424.37	0.197
**T4**	−290.86	32.41	300.35	189.69	1559.61	0.267
**T5**	−296.55	13.12	227.87	146.91	2959.94	0.114

## Data Availability

The original contributions presented in the study are included in the article; further inquiries can be directed to the corresponding authors.
